# Modelling the Effects of Prey Size and Distribution on Prey Capture Rates of Two Sympatric Marine Predators

**DOI:** 10.1371/journal.pone.0079915

**Published:** 2013-11-15

**Authors:** Chris B. Thaxter, Francis Daunt, David Grémillet, Mike P. Harris, Silvano Benvenuti, Yutaka Watanuki, Keith C. Hamer, Sarah Wanless

**Affiliations:** 1 School of Biology, University of Leeds, Leeds, United Kingdom; 2 Centre for Ecology & Hydrology, Bush Estate, Penuick, Midlothian, United Kingdom; 3 Centre d'Ecologie Fonctionnelle et Evolutive, UMR 5175 du CNRS, Montpellier, France; 4 Percy FitzPatrick Institute, DST/NRF Centre of Excellence, University of Cape Town, Rondebosch, South Africa; 5 Department of Biology, University of Pisa, Pisa, Italy; 6 Graduate School of Fisheries Sciences, Hokkaido University, Hakodate, Hokkaido, Japan; University of Waikato (National Institute of Water and Atmospheric Research), New Zealand

## Abstract

Understanding how prey capture rates are influenced by feeding ecology and environmental conditions is fundamental to assessing anthropogenic impacts on marine higher predators. We compared how prey capture rates varied in relation to prey size, prey patch distribution and prey density for two species of alcid, common guillemot (*Uria aalge*) and razorbill (*Alca torda*) during the chick-rearing period. We developed a Monte Carlo approach parameterised with foraging behaviour from bird-borne data loggers, observations of prey fed to chicks, and adult diet from water-offloading, to construct a bio-energetics model. Our primary goal was to estimate prey capture rates, and a secondary aim was to test responses to a set of biologically plausible environmental scenarios. Estimated prey capture rates were 1.5±0.8 items per dive (0.8±0.4 and 1.1±0.6 items per minute foraging and underwater, respectively) for guillemots and 3.7±2.4 items per dive (4.9±3.1 and 7.3±4.0 items per minute foraging and underwater, respectively) for razorbills. Based on species' ecology, diet and flight costs, we predicted that razorbills would be more sensitive to decreases in 0-group sandeel *(Ammodytes marinus)* length (prediction 1), but guillemots would be more sensitive to prey patches that were more widely spaced (prediction 2), and lower in prey density (prediction 3). Estimated prey capture rates increased non-linearly as 0-group sandeel length declined, with the slope being steeper in razorbills, supporting prediction 1. When prey patches were more dispersed, estimated daily energy expenditure increased by a factor of 3.0 for guillemots and 2.3 for razorbills, suggesting guillemots were more sensitive to patchier prey, supporting prediction 2. However, both species responded similarly to reduced prey density (guillemot expenditure increased by 1.7; razorbill by 1.6), thus not supporting prediction 3. This bio-energetics approach complements other foraging models in predicting likely impacts of environmental change on marine higher predators dependent on species-specific foraging ecologies.

## Introduction

The foraging ecology of marine higher predators has been the subject of intensive research in recent decades. Sophisticated animal-borne logging devices and ship-based surveys have helped identify important feeding areas [1]–[4], and diet sampling techniques have identified which prey predators are targeting [5]. However, the detailed interactions between predators and their prey, in particular prey capture rates, have rarely been quantified [6]–[8], yet are fundamentally important if we are to understand the role of environmental change on marine top predators. Furthermore, different species may show contrasting responses in prey capture rates to environmental conditions, associated with differences in foraging ecology. Quantifying prey capture rates is challenging because most marine predators feed out of sight of a land-based observer and many catch their prey underwater. Therefore, directly observing feeding events over prolonged periods is rarely possible. Prey capture rates can be assessed using animal-borne cameras that record foraging behaviour [9]-[11], or with gastric, oesophageal or magnetic loggers that record prey ingestion [12]–[14]. However, such methods are not readily applicable to small-sized species and/or those that eat small prey items. An alternative approach is to construct a bio-energetics model where information on daily energy requirements is used in conjunction with data on time activity budgets and diet to derive estimates of prey capture rate and estimate how such rates vary with prey availability [6]–[8].

Here, we use this latter approach for common guillemots (hereafter guillemot) *Uria aalge* and razorbills *Alca torda* to quantify prey capture rates. Both these species are wing-propelled pursuit-divers but their foraging behaviour differs with razorbills making predominantly short, shallow, V-shaped dives and guillemots typically making longer, deeper, U-shaped dives, although they do also make short, shallow dives [15]–[20]. During the breeding season, both species feed mainly on lipid-rich, shoaling fish which in the North Sea are predominantly lesser sandeels *Ammodytes marinus* or sprat *Sprattus sprattus* [15]–[21]. However, prey capture rates for the two species have not previously been quantified despite the fact that relevant data for an indirect approach are available in the literature. Furthermore, little is known as to how these species may respond when faced with environmental changes impacting the prey source. To address these concerns, we developed a bio-energetics model and estimated prey capture rates, expressed as energy gain over the time spent foraging and items caught per dive, for guillemots and razorbills breeding at a major colony in the North Sea. We then used this bio-energetics model to test predictions as to how these species may respond to changing environmental conditions. In our study area, two key components of prey availability have been changing: firstly, 0-group sandeels (fish hatched in the current year) have been getting smaller and thus the energy value of individual prey items has declined [22], [23]; and secondly, sandeel stock biomass has decreased [24], [25]. Optimal foraging theory predicts that predators seek to maximise their energy intake by obtaining the highest energy value food available or minimising the time spent acquiring prey [26], [27]. We therefore investigated three biologically plausible scenarios relating to changes in (1) prey size, (2) prey patch distribution, and (3) prey patch quality.

Under scenario (1) we modelled the consequences of decreasing size (and energy) of 0-group sandeels on prey capture rates assuming a proportional decrease in stock biomass. Diet data indicated that razorbills were more reliant on this prey type than guillemots and hence we predicted that razorbills would show greater sensitivity to declines in 0-group size. Under scenario (2) we modelled the effect of more dispersed prey patches to simulate the birds' response to a foraging environment requiring more flight. In this case, we predicted that guillemots would be more sensitive owing to their higher unit flight costs [16]. Under scenario (3) we compared prey capture rates of the two species when encounter rates with prey decreased, simulating the situation if fish density within prey patches declined. We predicted that the higher overall energetic demands of guillemots associated with larger body size [26], coupled with their obligate single prey loading method of chick provisioning, would make guillemots more sensitive than razorbills to increased time spent foraging as a result of decreased prey density.

## Methods

### Ethics statement

All fieldwork for this study had full ethical approval from the Ethics Committee of the Centre for Ecology & Hydrology. The Isle of May is a National Nature Reserve and all work on the island was approved under scientific and research licenses issued by Scottish Natural Heritage (SNH). Capture of birds at the breeding site, using an 8m telescopic pole with a noose or crook, and fitting of activity loggers to examine foraging behaviour were carried out under permits from SNH. Ringing activity was carried out under license from the British Trust for Ornithology. Water offloading to determine diets was carried out under project and personal licenses issued by the UK Home Office. Loggers were attached to the central back feathers with waterproof tape (Tesa AG, Hamburg, Germany), allowing loss through feather moult if not retrieved. Attachment took less than 15min, after which birds were released to the breeding site. Birds were recaptured after 1 to 10 days (usually 2 days) and the logger was removed. Birds typically returned to the breeding site and resumed normal brooding behaviour within 5 min of being released. Time spent at the colony during daylight hours did not differ between individuals with loggers and unequipped controls for either species, and no chick was lost during the period of deployment. Water offloading took less than 10 min, after which birds were released to the breeding site with no detectable adverse effects on their behaviour or breeding success.

### Model parameterisation

To estimate prey capture rates, data on the time activity budgets of adults, the diet of adults and chicks and the energetic requirements of adults and chicks are required [8], [21], [28].

### Time activity budgets

Fieldwork took place on the Isle of May, SE Scotland (56°11′N, 2°33′W) between 1999 and 2006. Breeding adults with 1–2 week old chicks (n = 25 guillemots, 11 razorbills) were captured from breeding ledges with an 8 m telescopic pole with a noose or crook, and tagged with data loggers. These loggers recorded activity from which we could distinguish nest site attendance, flight, presence on the sea surface and diving - see [16] for full details on deployment and data processing. The time spent in each activity was calculated over a 24 hour period (range 1–4 24 hour periods per bird). The number of dives was calculated for each 24 hour period. The time spent “foraging” was calculated as the summed duration of dives plus pauses on the sea surface between dives, and the time spent “underwater” was the summed duration of all dives [29], [30].

### Diet of adults and chicks

Adult diet was estimated from water-offloading chick-rearing adults - see [31] for full methodological details. Data on frequency of occurrence of prey types were available for 2003–2007 for guillemots and 2003 for razorbills. To investigate chick diet, three focal groups of adults (n = 22–33 pairs of guillemots and 6–17 pairs of razorbills) were observed from hides from dawn to dusk (03:00–23:00 British Summer Time; all prey are delivered during hours of daylight) on 2–4 days each year (1999–2006). Additional data were also collected during 2–3 hour watches made in 2005 and 2006. Fish were grouped into four size categories (very small, small, medium, and large – See Appendix S1 in File S1 for details of lengths) through comparison with adult bill length [32].

### Prey capture rate

We used a bio-energetics modelling approach to estimate the energy requirements of adults and chicks [8], [21], [28], and then estimated the prey capture rates necessary to balance these energy requirements. We used a Monte Carlo framework with 10,000 iterations encompassing the error associated with input parameters, by randomly sampling input parameters from empirically determined distributions or, where unavailable, assuming a 20% error [21]. However, a 20% error around beta regression coefficients resulted in unrealistic relationships, so we assumed an error of 2% in these cases.

The model first calculated the Daily Energy Expenditure (DEE) of adults for each 24 hour period by converting time activity budgets into energetic cost using estimates for activity-specific energetic costs taken from the literature [33]–[39]. We assumed that adults were in energy balance over the period and thus Daily Energy Intake (DEI) was equivalent to DEE plus the energy needed to warm ingested food, divided by the assimilation efficiency [39] - input parameters are given in Table 1 and Appendix S2 in File S1. The DEI of chicks was estimated from the all-day feeding watches by converting each prey type and size recorded to energy using regression equations [40] (see Appendix S1 in File S1), and then summing to give an overall DEI value per daily watch and year; means and standard deviations of DEI were calculated across all years from which a value was resampled on each MC run. Razorbills typically carry multiple prey items and the number of prey in loads is hard to quantify directly from visual observations. Therefore, we estimated the number of fish delivered per load based on the inverse relationship with prey size, following [21] - see Appendix S2 in File S1 for details. Finally we assumed that mates contributed equally to provisioning duties and thus 50% of the chick DEI was used to quantify adult energy requirements for the chick, which was then added to the respective adult component (see below).

**Table 1:** Parameter estimates used in the bio-energetics model for adult birds.

	Measure	Guillemot	Razorbill
Time allocation	Nest (%/day)	49.4±14.5	50.7±22.2
	Flight (%/day)	3.5±1.8	7.6±3.2
	Sea (%/day)	25.9±10.9	24.5±15.7
	Foraging (%/day)	21.2±8.1	17.3±8.4
	Underwater (%/day)	14.5±5.3	10.4±5.1
	No. adult dives/day	167±76	376±189
Other parameters	Mass (g)	908.4±53.4	582.9±26.0
	SST (°C)	11.7±1.0	12.6±0.5
	BMR (kJ/day)	390±78	311±62
	Assimilation efficiency (%)	77.52±1.60	78.97±1.71
	Food warming (kJ/day)	65±13	65±13
	Flight (W/kg)	92.6±18.5	71.2±14.2

See text and Appendix S2 in File S1 for metabolic relationships.

The bio-energetics model estimated: (1) number of prey caught per dive, (2) number of prey caught per minute spent foraging, and (3) number of prey caught per minute spent underwater separately for both adults and chicks. To estimate the number of prey per dive, the prey capture rate was first calculated as energy acquired per dive for each individual bird as follows:

Equation 1a: 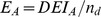



Equation 1b: 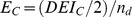
where, *E_A_*  =  energy per dive for the adult, *E_C_*  =  energy per dive for the chick, *DEI_A_*  =  daily energy intake of adult for self maintenance, (*DEI_C_*/2)  =  half the daily energy needed to provision the chick, and *n_d_* is number of dives. A value of *E_A_* and *E_C_* was then randomly selected from the distribution across all birds. Given that the diets of adults and chicks used different prey types and size distributions of individual prey (see Appendix S1 in File S1), we calculated the biomass proportion of each prey type in adult and chick diets. In the adult diet we converted the frequency of occurrence of prey items into relative energy proportions using the relationships for each prey item described in Appendix S2 in File S1. For guillemots, we used the mean frequency distribution across years from which a frequency proportion was sampled and then converted to energy proportions on each MC run (Appendix S3 in File S1). Six diet samples were obtained from adult razorbills, and all only contained remains of 0-group sandeels (Appendix S3 in File S1), hence adult diet of razorbills was assumed to be 100% 0-group sandeels. For the chick diet of both species, we used daily feeding rate and prey type to estimate the daily energy intake of different prey items and hence relative biomass proportions of prey types in the chick diet (Table 2 and Appendix S1 in File S1) on each MC run. The total number of prey needed to meet *E_A_* and *E_C_* was then calculated separately for adults and chicks as:

**Table 2:** (A) Mean prey species by frequency, energetic proportion, and size [31] for adults used in the bio-energetics model, and (B) prey species by frequency, energetic proportion for chicks used in the bio-energetics model.

A					
Adults	Measure	0-group sandeel	1+ group sandeel	Sprat	Gadid
Guillemot^a^	Proportional frequency (%)	36.5±20.6	8.4±8.7	31.3±20.9	23.8±18.0
	Proportional Energy (%)	14.6±16.5	8.7±6.2	75.4±18.6	1.4±1.5
	Size (mm)	52.5±9.4	96.6±8.7	88.8±11.0	25.0±8.7
	Energy of individual prey (kJ)^c^	2.72±1.77	19.88±6.73	45.97±25.70	0.47±0.56
Razorbill^a^	Proportional frequency (%)	100	0	0	0
	Proportional Energy (%)	100	0	0	0
	Size (mm)	52.5±9.4^b^	-	-	-
	Energy of individual prey (kJ)^c^	2.72±1.77	-	-	-

B					
Chicks^d^	Measure	0-group sandeel	1+ group sandeel	Sprat	Gadid
Guillemot	Proportional frequency (%)	1.0±0.7	26.4±13.6	70.6±15.7	2.0±1.4
	Proportional Energy (%)	0.1±0.0	17.2±8.7	82.1±9.3	0.7±1.1
Razorbill	Proportional frequency (%)	83.0±21.7	9.5±14.1	7.3±7.3	0.2±0.0
	Proportional Energy (%)	56.7±36.7	12.0±13.1	25.9±20.9	5.4±3.8

Table 2A: A division of 60 mm was chosen for 0-group sandeel and 1+ group sandeel based on fish collected from flight-netting puffins [22]. Proportions for guillemots are expressed as means across years of data collection – see Appendix S3 in File S1 for full data.

Table 2B: See Appendix S1 in File S1 for more information on decisions used on raw data from all-day watches to estimate prey proportions for chicks.

a Based on regurgitated samples from the Isle of May 2003 - 2007 [31].

b Using the same 0-group prey size as guillemots.

c Mean length value converted to energy [31].

d Information on the size of prey items deleivered to chicks are presented in Appendix S1 in File S1.

Equation 2a:
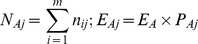



Equation 2b: 
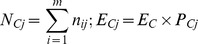



For adults (Equation 2a), using energy-length relationships for prey types, the algorithm iteratively sampled the number of prey *n_i_* for prey type *j*, for a particular biomass proportion of that prey type *P_Aj_* until *E_Aj_*, the proportion *P_Aj_* of the energy per dive *E_A_*, was met, i.e. *N_Aj_* had energy *E_Aj_*. The algorithm then summed the number of prey per dive for each prey type *j* (0-group sandeel, 1+ sandeel, sprat, and gadid), to give the total number of prey per dive *N_A_*, such that the number of prey sampled *N_A_* had energy *E_A_*. The same process was also repeated for chicks using Equation 2b, and then the total number of prey per dive for adults and chicks combined *N* was calculated as: *N_A_* + *N_C_*. The same process was carried out to estimate the number of prey per minute foraging and prey per minute underwater, by substituting *n_d_* in Equation 1 with *t_f_* (time spent foraging) and *t_u_* (time spent underwater), and then following the calculations through in Equation 2.

To test the sensitivity of the prey capture rate estimate to parameter error, we examined individual parameters whilst holding all others constant [21]. We only present sensitivity results for prey per dive, but results for the other prey capture currencies were quantitatively similar.

### Modelling consequences of poorer feeding conditions

#### Reduction in prey size (scenario 1)

We simulated a decrease in the size of 0-group sandeels (scenario 1) across the range of values reported in [23] from 70 to 30 mm. We assumed that the proportion of prey types in the diet and time activity budgets of guillemots and razorbills were unchanged from those used in the estimates of prey capture described above.

#### More dispersed prey patches (scenario 2) and reduced prey density within patches (scenario 3)

We assessed the influence of changing adult time budgets, and therefore DEE, as a likely response to changes in prey patch distribution and prey density within patches [28], [41]. In scenario 2, where prey patches were assumed to become more widely dispersed, birds were simulated to spend longer travelling between patches, and increase time spent foraging to acquire more energy to satisfy the cost of increased flight. In scenario 3, prey density within patches was assumed to decrease and foraging time within a patch was simulated to increase. In both scenarios the starting point was approximately the baseline daily activity budget (expressed as % time) for each species (Table 1 and Appendix S4 and S5 in File S1). Deteriorating foraging conditions were investigated by increasing time spent in flight and foraging (scenario 2), and increasing time spent foraging only (scenario 3), at the expense of time at the breeding site and resting on the sea surface. Under scenario 2 for both species, flight was increased and time on the sea decreased in increments of 2%, whilst foraging was increased at the expense of time at the nest in increments of 5%. In scenario 3, time spent foraging was increased in increments of 4% at the expense of time resting on the sea and at the breeding site, which were progressively decreased in increments of 2%. The upper limit for daily energy expenditure for vertebrates has traditionally been taken as 4 x BMR [28], [42], however, more recent work has suggested a higher estimate of 7 x BMR [43]. Therefore, when interpreting these results, we adopted this higher threshold – for more information see Appendix S2 in File S1. Using the bio-energetics model above, we calculated the DEE for all time activity budgets in the two scenarios. In order to present changes in relation to a basal metabolic rate and this potential metabolic ceiling, we expressed DEE as multiples of BMR.

### Statistical analyses

To assess inter-specific differences in time activity budgets we included a random effect of 24 hour period nested within bird ID, to test for the main effect of species, whilst allowing for repeated measures of 24 hour periods for individual birds. Significance was assessed through χ^2^ for models with non-normal errors and F-tests for models with normal errors. The significance of prey capture rates between species was assessed using Z-tests. For each paired species test, the differences in predicted values were divided by the standard error of the difference and compared against the Z distribution. All simulations were conducted using SAS 9.2 (SAS Institute Inc) and R 2.15.0 [44], and all means are presented ±1 standard deviation of the mean.

## Results

### Time activity budgets

Analysis of time activity budgets at the Isle of May for the two species indicated that daily activity differed in several ways (Appendix S4 and S5 in File S1). Razorbills spent significantly longer in flight than guillemots (1.8±0.8 hours per day compared to 0.8±0.4 hours per day, F_1,34_ = 15.0, P<0.001). Although on average razorbills made more dives than guillemots (376±189 dives per day compared to 167±76 dives per day, GLMM test of species, χ^2^_1_ = 11.3, P<0.001), there was no significant difference in the overall time spent foraging per day between the two species (guillemots: 5.1±1.9 hours per day; razorbills: 4.2±2.0 hours per day; F_1,34_ = 3.4, P = 0.073). However, guillemots spent significantly longer underwater than razorbills (3.5±1.3 hours per day compared to 2.5±1.2 hours per day; F_1,34_ = 14.6, P<0.001).

### Diet of adults and chicks

The frequency of occurrence of each prey type recorded in adult diet is presented in Appendix S3 in File S1. Adult guillemots took a range of prey, including 0-group and 1+ group sandeel, sprat, gadid such as saithe *Pollachius virens* and whiting *Merlangius merlangus*, goby species (Gobiidae), pipefish (Syngnathinae), and invertebrates (mainly crustaceans and polychaete worms). However, the majority of the diet was composed of sandeel, sprat and gadid, so subsequent modelling was restricted to these three prey types. The mean values across years are given in Table 2A. For guillemot, 0-group sandeels were the most frequently recorded item in the diet (36.5±20.6%), but sprats made up the majority of diet energetically (75.4±18.6%). Data for razorbills indicated that the only prey consumed was 0-group sandeels (Appendix S3 in File S1).

Diet composition of guillemot and razorbill chicks expressed as frequency of occurence and biomass is shown in Table 2B. Both the species (Binomial GLM, χ^2^_2_ = 147.3, P<0.001) and sizes (χ^2^_2_ = 259.3, P<0.001) of prey brought in differed. Most items delivered to chicks by adult guillemots were sprats (70.6±15.7% feeds), with an average length of 103.2±4.9 mm. The next most abundant prey item was 1+ sandeels (26.4±13.6%, mean length 105.9±9.0 mm). In contrast, the majority of feeds delivered to razorbill chicks comprised multiple prey items (73.5±13.4%); predominantly 0-group sandeels (83.0±21.7%, mean length 49.1±2.9 mm), with loads composed of either 1+ sandeels (9.5±14.1%, 73.1±46.6 mm) or sprats (7.3±7.3%, 71.1±12.1 mm) making up most of the remainder. Only a small proportion (63 feeds, <3%) were loads comprising multiple species and/or sizes. Expressing chick diet in terms of energy indicated that sprats made up 82.1±9.3% of the diet of guillemot chicks, whereas 0-group sandeels made up over half of the diet of razorbill chicks (56.7±36.7%, Table 2).

### Prey capture rate

The prey capture rate of adult guillemots was estimated as 1.5±0.8 items per dive, 0.8±0.4 items per minute foraging and 1.1±0.6 items captured per minute underwater (Table 3). Equivalent values for adult razorbills were 3.7±2.4 prey per dive, 4.9±3.1 and 7.3±4.0 prey per minute foraging and underwater, respectively. Prey capture rates of razorbills were significantly higher than guillemots (Z = 66.5, Z = 94.3, Z = 110.0, for prey per dive, minute foraging, and minute underwater respectively, P<0.001 in all cases), largely due to the greater reliance on smaller prey items (Table 3). The bio-energetics model was most sensitive to changes in time activity budgets (CV = 0.366 and 0.645 for guillemots and razorbills respectively, Table 4), but was also sensitive to the sizes of fish consumed by adults, in particular 0-group sandeel (CV = 0.326 for guillemots and 0.423 for razorbills), and sprat (CV = 0.170 for guillemots and 0.153 for razorbills). Prey proportions in the adult diet also influenced the model (CV = 0.111 for guillemots and 0.079 for razorbills).

**Table 3:** Prey capture rates from the bio-energetics model from 10,000 MC simulations assessed under a standard diet for both species (prey sizes, prey proportions).

	Prey capture rate per		
Species	Dive	Minute foraging	Minute underwater
Razorbill	3.7±2.4	4.9±3.1	7.3±4.0
Guillemot	1.5±0.8	0.8±0.4	1.1±0.6

**Table 4:** Sensitivity analysis of parameters used in Monte Carlo simulation, shown here for prey per dive.

	Coefficient of variation (CV)		Calculation	
Variable	Guillemot	Razorbill	Used in	Reference
Mass (kg)	0.038	0.018	Diving metabolic rate; W/kg∼kJ	[36]
a	0.004	0.014	Diving (kJ) = 10?(a+b*log10(Mass))	[36]
b	0.068	0.049	Diving (kJ) = 10?(a+b*log10(Mass))	[36]
SST (°C)	0.010	0.004	Sea surface temperature	This study
a	0.002	0.002	Sea (W/kg) = a-(b*SST (°C))	[37]
b	0.007	0.004	Sea (W/kg) = a-(b*SST (°C))	[37]
Assimilation (%)	0.023	0.018	Adult daily energy intake (kJ/day)	[39]
Food warming (kJ/day)	0.010	0.012	Adult daily energy intake (kJ/day)	[39]
BMR (kJ/day)	0.062	0.065	Nest (kJ/day) = 2*BMR (kJ/day)	[35]
**Adult time budget (%/day)**	**0.366**	**0.645**	Individual time budgets of adults	This study
Adult diet proportions (%)	0.111	0.079	Proportion of prey in adult diet	[31], This study
Flight (W/kg)	0.035	0.049	Flight metabolic rate	[33], [34]
a	0.012	0.010	Sandeel (kJ) = a*length (cm)?b	[40]
b	0.072	0.069	Sandeel (kJ) = a*length (cm)?b	[40]
a	0.005	0.005	Sprat (kJ) = a*length (cm)?b	[40]
b	0.056	0.046	Sprat (kJ) = a*length (cm)?b	[40]
a	0.001	0.001	Gadid (kJ) = (a* length (cm)?b)*c	[40]
b	0.004	0.003	Gadid (kJ) = (a* length (cm)?b)*c	[40]
c	0.001	<0.001	Gadid (kJ) = (a* length (cm)?b)*c	[40]
**Sandeel 0-group (mm)**	**0.326**	**0.423**	Size of prey in adult diet	[31]
Sandeel 1+ group (mm)	0.045	0.052	Size of prey in adult diet	[31]
**Sprat size (mm)**	**0.170**	**0.153**	Size of prey in adult diet	[31]
Gadid size (mm)	0.017	0.018	Size of prey in adult diet	[31]
Chick DEI (kJ/day)	0.001	0.007	Energy intake per chick per day	This study
Chick diet proportions (%)	0.001	0.028	Proportion of prey in chick diet	This study
Sandeel 0-group (mm)	<0.001	0.015	Size of prey in chick diet	This study
Sandeel 1+ group (mm)	0.001	0.052	Size of prey in chick diet	This study
Sprat size (mm)	0.001	0.012	Size of prey in chick diet	This study
Gadid size (mm)	<0.001	<0.001	Size of prey in chick diet	This study

The three highest CV values and hence the variables giving most influence in calculation of prey capture rates, are highlighted in bold for both species.

### Reduction in prey size (scenario 1)

**Figure 1:**
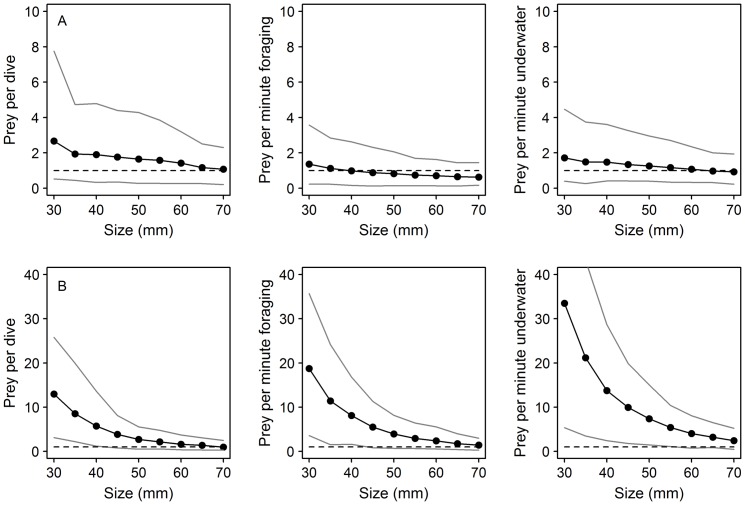
Influence of changing prey size on prey capture rates for guillemots and razorbills. Example for (A) guillemots and (B) razorbills, illustrating the influence of a decrease in 0-group sandeel size (prey quality) on prey capture rates; this scenario assumes both species still had the same proportion of prey items in their diets and other prey sizes did not decrease in size.

For guillemots, prey capture rates increased non-linearly as 0-group sandeel size decreased, reaching values of 2.7±2.0 prey per dive (1.4±1.0, and 1.7±1.1 prey per minute foraging and minute underwater, respectively) for 0-group that were 30mm in length (Fig. 1A). For razorbills, the increase in prey capture rates as 0-group size decreased was even more pronounced (13.0±6.2 prey per dive, 18.7±8.5 and 33.5±15.2 prey per minute foraging and underwater, respectively) when 0-group length was reduced to 30mm (Fig. 1B).

### More dispersed prey patches (scenario 2) and reduced prey density within patches (scenario 3)

**Figure 2:**
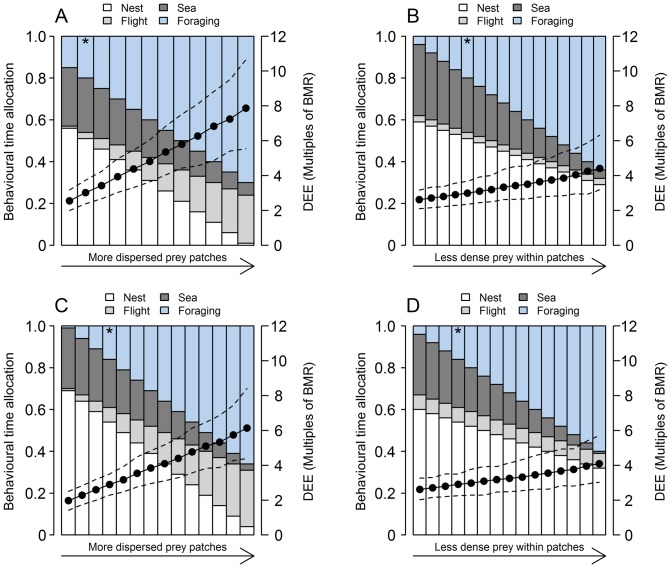
Influence of prey patch dispersion and density on energy expenditure for guillemots and razorbills. Simulations of proportional daily time budgets and daily energy expenditure (DEE) for guillemots (A, B) and razorbills (C, D) where: (1) prey becomes more patchily distributed requiring more flight time between patches and more foraging time to meet energetic needs (A, C); and (2) prey decreases in density within patches, requiring more foraging time, but distribution is unchanged (B, D). Asterisks indicate the proportion of time activity budget which is the mean across all recoded activity budgets of birds of each species, respectively (see Appendix S4 and S5 in File S1).

Model results for scenarios when prey patches were more dispersed, indicated a steeper increase in daily energy expenditure for guillemots than razorbills (Fig. 2), due to higher flight costs in the former (Table 1). Therefore, guillemot DEE increased from 996±119kJ for a simulated foraging environment where patches were close together (1% flight, 15% foraging), to 3079±487 kJ where patches were widely dispersed (23% flight, 70% foraging). Comparing razorbills in the same way indicated that DEE increased from 807±93 kJ (5% flight, 11% foraging) to 1909±324 kJ (27% flight, 66% foraging). For scenario 2, differences in DEE over the range of prey patch dispersion modelled were therefore greater by a factor of 3.1 for guillemots and 2.4 for razorbills. In contrast, when prey density within patches was reduced and foraging time but not flight time was increased, guillemot DEE increased by only 705 kJ, from 1017±109 kJ (3% flight, 4% foraging) to 1722±312 kJ (3% flight, 68% foraging), whereas razorbill DEE increased by only 460 kJ from 815±97 kJ (7% flight, 4% foraging) to 1275±207 kJ (7% flight, 60% foraging). For scenario 3, differences in DEE over the range modelled were therefore greater by a factor of 1.7 for guillemots and 1.6 for razorbills.

## Discussion

This study used a bio-energetics approach parameterised with data on foraging behaviour and adult and chick diet, to obtain estimates of prey capture rates of common guillemots and razorbills, two key members of the North Atlantic seabird community. We then used these results to explore how these species might respond to a range of biologically plausible changes in the quality and distribution of one of their main prey species, the lesser sandeel. Based on our previous knowledge of flight energetics and chick provisioning strategy, and empirical data on adult and chick diet collected during the study, we predicted that the two species would be affected by different aspects of prey availability. Specifically we predicted that (1) razorbills would be more sensitive to reduced prey size, (2) guillemots would be more sensitive to more patchily distributed prey, and (3) guillemots would be more sensitive to prey patch quality in terms of reduced density of fish within a shoal. Predictions 1 and 2 were both supported by the model results. However, modelled response rates to simulated decreases in patch quality were similar in the two species and thus were not consistent with our prediction that guillemots would be more sensitive than razorbills to variation in this aspect of prey availability.

### Inter-specific differences in prey capture rates

For both guillemots and razorbills, the chick is only on the cliff nest site for about 21 days, and is then taken to sea by the male parent to complete the main part of its growth [45]. Consequently, *ca*. 88–94% of the daily food requirements while the chick is in the colony is used to meet adult requirements, whereas only *ca*. 6–12% is used to feed the chick (11.8% for guillemots and 6.2% for razorbills). Estimated prey capture rates in terms of fish caught per dive are therefore influenced more by adult than chick diet. However, data on adult diet of both common guillemots and razorbills are extremely limited. The additional data for guillemots presented here support earlier results for birds at this colony and indicate that parents select larger fish to feed to the chick than they eat themselves [31]. Only six adult diet samples were obtained for razorbills, but these are the first for this species on the Isle of May, and we are not aware of equivalent data from other colonies. These samples, like those for guillemots, indicate that razorbills select larger prey for the chick than they take themselves. Seabirds face different contraints when self-feeding compared to provisioning the young, which may result in differences in diets [46]. Concurrent studies of adult and chick diets are relatively scarce [5], but similar parent/chick prey size disparities to those reported here have been found in species that transport prey to the chick in the bill [47], [48]. Assuming that dietary results for our study are robust, the model indicated that mean prey capture rates were 1.5 items per dive for common guillemot, and 3.7 items per dive for razorbills. Given that some dives may be unsuccessful these values suggest that both species, but particularly razorbills, capture more than one prey item during a dive and swallow prey underwater. Razorbills consistently make more dives per trip than guillemots [16] and the present study suggests that this difference is maintained throughout a 24 hour period. In terms of prey capture rates per minute foraging and underwater, differences in activity budgets, in particular time spent underwater per day, resulted in prey capture rates for razorbills being four times higher that those of guillemots. Several other studies have also concluded that multiple prey captures per dive are common. For example, European shags *Phalacrocorax aristotelis* feeding on sandeels were estimated to catch on average between 1.4 and 6.0 fish per dive [49], and Adelie penguins *Pygoscelis adeliae* feeding on krill captured multiple items per dive up to rates of two krill per second [50]. However, obtaining direct information on success rates of dives for relatively small bodied species like alcids remains challenging. A pilot study using gastric loggers with guillemots on the Isle of May, suggested around 30% of dives were successful [51]. Applying this figure to our results suggests prey capture rates attained on successful dives may be of the order of 5.0 prey per dive for guillemots and 12.3 prey per dive for razorbills.

### Prey capture responses under changing environmental conditions

Relationships between body length and energetic value (kJ) of many fish species are non-linear [40]. Therefore, to maintain DEI if the average size of prey declines, predators can respond by showing a non-linear increase in prey capture rate. In our scenario, simulating a decline in the length, and hence energy value, of 0-group sandeels, the response curve for razorbills was markedly steeper than for guillemots. This supported our first prediction that razorbills on the Isle of May would be more sensitive to this component of prey availability due to their greater reliance on this age class. In addition to declines in the length-at-age of sandeels in the seas around the Isle of May [22], [23], there has also been a decline in biomass [24], [25]. The underlying cause is uncertain, but plausible scenarios are for shoals to become more widely dispersed, or that density of fish within a shoal declines. We simulated the potential effects of both these scenarios on daily energy budgets of guillemots and razorbills during chick rearing. In both cases we anticipated that guillemots would be the more sensitive species due to their higher wing loading and hence flight costs, and greater body mass and hence higher absolute daily energy requirements. This expectation was supported in the scenario where prey patches were simulated to become more widely dispersed, requiring increased flight time between patches. The DEE increased markedly in both species but the response curve was steeper for guillemots than razorbills (upper value for guillemots was 8 times BMR compared to 6 times BMR for razorbills, Fig. 2A and C). These results were not driven by unusual time budgets of these species at the Isle of May, since activity budgets were broadly similar to those recorded elsewhere (see Appendix S2 in File S1 for a review). In contrast, responses of both species to lower encounter rates within patches were similar and over the range of values simulated, although DEE did increase, birds were able to increase time spent foraging when within-patch prey density decreased, without apparently impacting on DEE (as defined by no overlap of 95% confidence intervals of modelled values with energetic ceiling of 7 x BMR [43]; Fig. 2B and D). The lack of support for a marked difference between guillemots and razorbills to changes in prey encounter rates, contrasts with a previous study based on observations of birds at sea and concurrent acoustic data on prey density, that found that Atlantic puffins were on average, associated with sparser prey patches than guillemots [26]. This spatial segregation was attributed to differences in body size (guillemots are approximately three times heavier than puffins) and hence differences in absolute amounts of prey required per day, which was suggested as a mechanism for promoting co-existence. It is possible that our measure of foraging duration in prey patches was too coarse to detect finer-scaled predator prey-interactions related to less dense prey patches or prey patches of overall lower energetic value, both of which could be attributed to prey patch “quality”. Such questions can be tackled further through more complex modelling approaches [52], [53]. For instance in a recent study [53] three predators with different foraging constraints were compared: black-legged kittiwake *Rissa tridactyla*, northern fur seal *Callorhinus ursinus*, and Brunnich's guillemot *Uria lomvia*; in all cases habitat use was most strongly predicted by prey patch characteristics (e.g. depth and local prey density). However, all species were similarly linked by patchiness of prey rather than by the distribution of overall biomass or numerical abundance [53]. These findings accord with our study system where birds were apparently most sensitive to patchiness of prey and individual prey size. Furthermore, broad-scale low density patches may contain high density smaller-scale ones, that may induce scale-dependent shifts in movement patterns (area restricted search, ARS, behaviours) [54], [55]. To efficiently acquire prey, a recent study found that common guillemots in Newfoundland exhibited small-scale (2 km) ARS behaviour, flight and foraging movements indicative of Brownian motion, and deterministic memory-based search behaviour, reflecting the distribution of a key prey source – capelin *Mallotus villosus* [55]. Therefore, the micro-scale, three-dimensional characteristics of prey patches may be crucial for determining how they are perceived and exploited by air-breathing predators [56]–[58].

### Methodological development

Our bio-energetics approach has highlighted differences in prey capture rates and energetic constraints in two wing-propelled, pursuit-diving seabirds associated with morphometrics and diet which could be difficult to detect using traditional foraging models. The approach could be further refined by incorporating empirical data on the spatial aggregation of the prey field. Guillemots and razorbills on the Isle of May are known to show some segregation in feeding areas and marked segregation in feeding depths [16]. Likewise, a recent study at a colony in Greendland found that common guillemots and razorbills fed in similar areas but segregated vertically with guillemots diving deeper than razorbills [59]. It would be highly informative to combine studies using bird-borne data loggers to record detailed foraging behaviour with concurrent acoustic data on prey density [53], [55], as well as dietery information [59]. Such data would be useful to understand how landscape-properties and large-scale prey spatial distributions may affect prey detection patterns in seabirds [52], [55]. With deteriorating feeding conditions, at some point an energetic threshold will be reached above which an individual cannot operate sustainably. In K-selected species such as guillemots and razorbills, adults are predicted to prioritise self-feeding over provisioning the chick when conditions deteriorate [60]. Chick desertion by guillemots when feeding conditions have been severe has been recorded recently on the Isle of May [61]. However, on the scale presented in Fig. 2, the threshold at which this may occur cannot currently be determined without further direct metabolic investigation at this colony.

### Conclusions

This study has shown the potential of a bio-energetics approach to model inter-specific differences in prey capture rates in relation to changing environmental conditions. Our model highlights how two sympatric-breeding species, the common guillemot and razorbill, vary in sensitivity to different aspects of prey availability due to relatively small differences in diet and foraging behaviour. In our study system, razorbills appear more sensitive than guillemots to changes in prey size whilst guillemots appear more sensitive to changes in the distribution of prey. The bio-energetics approach we have applied here complements rather than replaces other types of foraging model, for instance individual-based models, and serves to highlight the usefulness of bird-borne logging devices in relation to energetics of individuals. Work currently being carried out at other colonies in the North Atlantic will potentially allow assessment of prey capture rates across a wider range of environmental conditions and prey types. Given the changes that are known to be occurring in many prey populatons due to climate change and fisheries, information on predator-prey interactions such as these are vital in order to better understand, and in turn safeguard, internationally important seabird populations and the wider marine environment.

## Supporting Information

File S1 **Appendix S1**, Prey species delivered by adults to chicks during feeding watches at the Isle of May (1999-2006). **Appendix S2**, The bio-energetics model and energetic relationships from the literature. **Appendix S3**, Frequency of prey types in adult diets at the Isle of May (2003-2007) obtained through water offloading. **Appendix S4**, Time activity budgets for all common guillemots included in this study. **Appendix S5**, Time activity budgets for all razorbills included in this study.
(DOCX)
