# Supplementary material for: Modelling the Effects of Prey Size and Distribution on Prey Capture Rates of Two Sympatric Marine Predators
Source: PLoS One. 2013 Nov 15;8(11):e79915. doi: 10.1371/journal.pone.0079915 (PMC3829866; doi:10.1371/journal.pone.0079915)
Supplement: File S1 — Appendix S1, Prey species delivered by adults to chicks during feeding watches at the Isle of May (1999-2006). Appendix S2, The bio-energetics model and energetic relationships from the literature. Appendix S3, Frequency of prey types in adult diets at the Isle of May (2003-2007) obtained through water offloading. Appendix S4, Time activity budgets for all common guillemots included in this study. Appendix S5, Time activity budgets for all razorbills included in this study. (DOCX) [file pone.0079915.s001.docx]

**File S1. Supporting information.**

**Appendix S1. Prey species delivered by adults to chicks during feeding watches at the Isle of May (1999-2006).**

Data are presented by frequency, energetic proportion, and size, highlighting decisions that were made on the raw data and subsequent recorded mean size and energy (see Appendix S2 in File S1) of prey. A division of 60mm was chosen for separating 0-group sandeels, and 1+ group sandeels based on fish collected from flight-netting puffins [1]. These sizes and proportions were used in the chick component of the prey capture rate algorithm.

| **Seabird species** | **Measure** | **0-group sandeel** | **1+ group sandeel** | **Sprat** | **Gadid** |
| --- | --- | --- | --- | --- | --- |
| **Guillemot** | Proportion (frequency %) | 1.0±0.7 | 26.4±13.6 | 70.6±15.7 | 2.0±1.4 |
|  | Proportion (kJ %) | 0.1±0.0 | 17.2±8.7 | 82.1±9.3 | 0.7±1.1 |
|  | Very small size (mm)^1^ | 60.0±0 | - | 70.0±14.1 | - |
|  | Small size (mm)^1^ | - | 93.3±5.8 | 96.7±5.8 | 50.0±0 |
|  | Medium size (mm)^1^ | - | 123.3±5.8 | 123.3±11.6 | 80.0±0 |
|  | Large size (mm)^1^ | - | 143.3±5.8 | 150.0±0 | 120.0±0 |
|  | Overall size (mm)^2^ | 60.0±0 | 105.9±9.0 | 103.2±4.9 | 71.9±11.5 |
|  | Overall energy of prey (kJ) | 3.7±0.2 | 29.5±15.8 | 83.9±46.4 | 15.2±12.6 |
| **Razorbill** | Proportion (frequency %) | 83.0±21.7 | 9.5±14.1 | 7.3±7.3 | 0.2±0.0 |
|  | Proportion (kJ %) | 56.7±36.7 | 12.0±13.1 | 25.9±20.9 | 5.4±3.8 |
|  | Very small size (mm)^1^ | 40 | - | 50 | - |
|  | Small size (mm)^1^ | 50 | - | 60 | 90 |
|  | Medium size (mm)^1^ | - | 70 | 80 | 110 |
|  | Large size (mm)^1^ | - | 80 | 90 | - |
|  | Overall size (mm)^2^ | 49.1±2.9 | 73.1±46.6 | 71.1±12.1 | 98.0±10.9 |
|  | Overall energy of prey (kJ) | 1.9±0.3 | 7.7±1.8 | 24.6±13.6 | 25.0±7.7 |
|  | Overall energy per load (kJ) | 15.9±6.5 | 13.4±10.3 | 40.6±32.1 | 46.6±23.4 |
|  | Very small number per feed^3^ | 10.5±4.6 | - | 5.4±1.9 | - |
|  | Small number per feed^3^ | 8.2±8.2 | - | 1.6±1.3 | 2.2±1.2 |
|  | Medium number per feed^3^ | - | 1.6±1.5 | 1.1±1.1 | 1.0±0 |
|  | Large number per feed^3^ | - | 1.3±0.8 | 1.2±0.7 | - |
|  | Overall number per feed^3^ | 8.4±3.4 | 1.5±1.3 | 1.6±1.7 | 2.0±1.2 |

^1^ Defined sizes for each prey type and size based on prey collected on breeding ledges, from fish dropped by puffins, and subjective decisions

^2^ The mean observed overall size, based on decisions made under 1 above

^3^ The mean observed number of prey per feed for razorbill for each prey type, and an overall mean (independent of any decisions on prey size)

**Appendix S2. The bio-energetics model and energetic relationships from the literature.**

We calculated daily energy expenditure (*DEE_A_*) and hence daily energy intake (*DEI_A_*) of adults as

Equation 1:

Equation 2:

where, *fw* = energy expended warming the food ingested, and *AS* = gut assimilation efficiency; proportional time activity are given as: *propFL*, *propFO*, *propSE*, and *propNE*, defined as the proportion of time per 24 hours spent in flight, foraging (diving plus interdive pause), on the sea surface and at the nest, respectively. Proportions of time activity were calculated directly from activity budgets of individuals, and were available for 1-4 (typically 1-2) 24 hour sections per bird. We defined *FL.kJ*, *FO.kJ*, *SE.kJ*, and *NE.kJ* as the metabolic rate (kJ/day) of individuals during activities of flight, foraging, on the sea surface and at the nest, respectively. Assimilation values were taken from the literature [2] and were given as 77.52±1.60% for guillemots and 78.97±1.71% for razorbills. The energy expended on the sea surface, was defined as:

Equation 3: $SE.Wkg=17.39-\left(0.6\times SST\right)$[3]

Equation 4: $SE.kJ=\left(\left(SE.Wkg\times Mass\right)\times60\times60\times24\right)/1000$

where, *SE.Wkg* = metabolic rate (*W/kg*) during periods on the sea surface, *SST* = sea surface temperature recorded directly for 18 guillemots and 6 razorbills from the compass logger temperature sensor, and *Mass* = body mass (kg) of the individual either measured directly, or sampled from the distribution of observed masses in this study. Direct mass measurements were available for only eight guillemots and six razorbills that were tagged in this study; therefore we also calculated a mean mass including other data that were available from separate samples of birds collected over the same time period; mean masses were thus given as 908.4±53.4g for guillemots (n = 25) and 582.9±26.0g razorbills (n = 20). Temperature during phases on the sea surface was calculated for each bird where possible; however, when temperature was unknown (see above), we took a random sample from the distribution across all birds and time sections (guillemot, mean: 11.7±1.0ºC, range: 10.1-13.1ºC; razorbill, mean: 12.6±0.5ºC, range: 11.8-13.9ºC). We estimated the metabolic rate (kJ/day) during diving underwater (*DI.kJ*) as:

Equation 5: [4]

To obtain “foraging metabolic rate” (*FO.kJ*), we added the diving metabolic rate (*DI.kJ*) to a calculated metabolic rate for inter-dive periods on the sea surface, thus:

Equation 6: $FO.kJ=DI.kJ+\left(PropUnder\times SE.kJ\right)$

where *PropUnder* = the proportion of time spent underwater per day. For periods of flight, we calculated *FL.kJ* (metabolic rate measured in kJ/day) as:

Equation 7: $FL.kJ=\left(\left(FL.Wkg\times Mass\right)\times60\times60\times24\right)/1000$

where *FL.Wkg* = metabolic rate (W/kg) of energy expenditure during flight using Program FLIGHT [5], [6], given as 92.6W/kg for guillemots, and 71.2W/kg for razorbills. Finally, we defined the metabolic rate of energy expenditure at the nest (*NE.kJ*) as:

Equation 8: $NE.kJ=2\times BMR$

For guillemots and razorbills, basal metabolic rate (BMR) [7] was given as 390kJ/day and 311kJ/day, respectively. For estimation of the number of prey needed to meet energetic needs (see Equations 1 and 2 in the main article), we calculated the energy of fish from length-energy (kJ) relationships [8]:

Equation 9: $Sandeel (kJ)=0.0081\times length (cm)^{3.427}$

Equation 10: $Sprat (kJ)=0.0096\times length (cm)^{3.845}$

Equation 11: $Gadid (kJ)=\left(0.00608\times length (cm)^{3.15}\right)\times3.5$[8]

Only prey species where published energetic values were available could be used for modelling, hence we could not include information for gobies, invertebrates, or other species that have been recorded in adult diets (Appendix S3 in File S1). For calculation of daily energy intake of chicks (*DEI_C_*), we used the mean daily energy intake calculated directly from all-day watches. For individual feeds, the energetic value was calculated as the number of prey (guillemot = 1, razorbill >=1) multiplied by the energetic content of the respective prey items. This calculation used the same fish length-energy relationships as stated in equations 9, 10 and 11. Razorbills typically carry multiple prey items and the exact number of prey in loads is hard to quantify from visual observations. However, typically the number of fish is inversely related to the unit prey size and accordingly, we assumed that loads containing very small, small, medium and large fish comprised 10, 6, 4 and 2 individual prey respectively [9]. The *DEI_C_* was calculated by summing all feeds for individual chicks per watch and then averaging across all chicks for that watch. Averages were then taken to give one value and standard deviation of *DEI_C_* across all years, from which a random value could be drawn (mean, guillemot: 266±79kJ/day, mean razorbill: 102±37kJ/day); comparable data from other colonies are lacking for razorbills, but values of daily energy intake for guillemot chicks are similar to those at the Isle of May [1], [10]. The randomly sampled *DEI_C_* was then divided by 2 on the assumption that parents contributed equally to chick provisioning, and a value for the energy per dive, per minute foraging and per minute underwater was calculated. This value represented the energy acquired per currency by the adult to meet the chick’s need. A similar process to adult DEI (*DEI_A_*) was then used to sample the proportion and size of chick prey items, using all day watches, to meet this energy per currency value (see Equations 1 and 2 in the main article).

***Basal metabolic rate***

We used multiples of Basal Metabolic Rate (BMR) as a metric for simulations of predator responses to changes in the prey base. Previously, a value of 4 x BMR was considered an upper metabolic ceiling in birds [10], [11], prolonged expenditure beyond which was thought to be unsustainable [10]. However, several studies have concluded that the 4 x BMR ceiling may be too low, with values above this often reported [2], [12], [13]. A higher value of 7 x BMR has been described for vertebrates [14], and was therefore taken as representative for guillemots and razorbills in this study, as used elsewhere [15]. In our simulations the baseline condition (shown by asterisks in Fig 2) suggests birds may be operating at or just below 4 x BMR, which is similar, albeit slightly lower than has been recorded elsewhere for Brunnich’s guillemots using doubly labelled water (5.7 x BMR) [15], [16].

***Assumption of energy balance***

We assumed that adult birds were in energy balance whilst rearing chicks. However, the body mass of adult common guillemots is known to decrease between incubation and chick-rearing [17], [18], as also seen for Brunnich’s guillemots. These changes have been attributed to increasing seasonal demands in provisioning chicks [19], or adaptive responses to reducing flight costs and improving diving performance [20]. Adult mass loss for common guillemots over chick-rearing (from 1 to 25 day old chicks) is estimated as 97g [18]. However, the sensitivity analysis suggested our bio-energetics model was not sensitive to mass (Table 4). The effect of the energy balance assumption on our conclusions was therefore considered to be negligible.

***Time activity budgets – a comparison to other colonies***

The activity data for the Isle of May in this study was broadly similar to other studies of guillemots [21], [22-24] and razorbills [25], [26], [27], [28]. The mean time spent at the colony per day was 49.2% for guillemot, similar to previous studies (range, 30.3-71.0%), and for razorbills, our value of 48.9% compared well with a value of 50% recorded at Grӕsholmen in the Baltic Sea [25]. The time spent in flight for guillemot was 3.5%, at the lower end of the recorded range (range, 3.2-7.7%). For razorbills, no other information was available on the time spent in flight from other studies to compare with our mean figure (7.8% per day). However, for razorbills studied at the Isle of May over the same time period, 15.6% time was spent in flight per foraging trip, at the upper range seen across other studies (7-15%). The time spent underwater per day, 14.5% (23.5% including surface pauses) and 8.0% (21.3% including surface pauses), matched those reported elsewhere for both guillemots (3.9-16.7%) and razorbills (2.8-13.1%). The similarity of these figures to existing studies gives further weight to the conclusions reached for scenarios 2 and 3. Adults may alter their time budgets in response to changing environmental conditions, seen through reductions in time spent on the sea surface and increases in foraging activity [24] – these known behaviours were reflected in our decisions for alterations of time activity budgets when testing scenarios 2 and 3.

**Appendix S3**. **Frequency of prey types in adult diets at the Isle of May (2003-2007) obtained through water offloading.**

Data are shown for (A) Common guillemots (2003-2005), (B) Common guillemots (2006 and 2007) and (C) Razorbills (2003). Information is presented as the number of samples containing remains, otoliths, and percentage (%) with prey. For the purposes of Monte Carlo simulations, the total number with remains or otoliths was tallied to give a compositional value for each of the following prey types: 0-group sandeel, 1+ sandeel, sprat, and gadid. Other prey types could not be included because they had no known energetic relationships to prey size (see also Table 2).

| **A** |  | | |  | | |  | | |
| --- | --- | --- | --- | --- | --- | --- | --- | --- | --- |
| **Guillemot** | **2003** |  |  | **2004** |  |  | **2005** |  |  |
| **Prey type** | **Remains** | **Otoliths** | **% with prey** | **Remains** | **Otoliths** | **% with prey** | **Remains** | **Otoliths** | **% with prey** |
| Sandeel all | 59 | 44 | 95 | 6 | 6 | 75 | 3 | 2 | 30 |
| Sandeel (0-group) | 35 | 35 | 56 | 5 | 5 | 63 | 2 | 2 | 20 |
| Sandeel (1+ group) | 14 | 14 | 23 | 1 | 1 | 13 | 0 | 0 | 0 |
| Sprat | 14 | 6 | 23 | 5 | 1 | 63 | 1 | 1 | 10 |
| Goby | 13 | 6 | 21 | 0 | 0 | 0 | 2 | 2 | 20 |
| Gadid | 10 | 7 | 16 | 0 | 0 | 0 | 3 | 3 | 30 |
| Pipefish | 0 | 0 | 0 | 0 | 0 | 0 | 0 | 0 | 0 |
| Invertebrates | 13 | n/a | 21 | 1 | n/a | 13 | 4 | n/a | 29 |
| No. with prey | 62 | 48 | - | 8 | 8 | - | 10 | 5 | - |
| No. additional invertebrates | 0 | n/a | - | 0 | n/a | - | 4 | n/a | - |
| Total birds sampled | 62 |  | - | 17 | - | - | 19 | - | - |
| **B** |  |  |  |  |  |  |  |  |  |
| **Guillemot** | **2006** |  |  | **2007** |  |  |
| **Prey type** | **Remains** | **Otoliths** | **% with prey** | **Remains** | **Otoliths** | **% with prey** |
| Sandeel all | 0 | 0 | 0 | 8 | 7 | 47 |
| Sandeel (0-group) | 0 | 0 | 0 | 7 | 7 | 41 |
| Sandeel (1+ group) | 0 | 0 | 0 | 2 | 2 | 12 |
| Sprat | 11 | 1 | 65 | 3 | 3 | 18 |
| Goby | 2 | 2 | 12 | 0 | 0 | 0 |
| Gadid | 7 | 7 | 41 | 4 | 3 | 24 |
| Pipefish | 2 | 0 | 12 | 1 | 0 | 6 |
| Invertebrates | 11 | n/a | 61 | 4 | n/a | 22 |
| No. with prey | 17 | 10 | - | 17 | 9 | - |
| No. additional invertebrates | 1 | n/a | - | 1 | n/a | - |
| Total birds sampled | 21 | - | - | 21 | - | - |
| **C** |  |  |  |  |  |  |
| **Razorbill** | **2003** |  |  |
| **Prey type** | **Remains** | **Otoliths** | **% with prey** |
| Sandeel all | 6 | 4 | 100 |
| Sandeel (0-group) | 6 | 4 | 100 |
| Sandeel (1+) | 0 | 0 | 0 |
| Sprat | 0 | 0 | 0 |
| Goby | 0 | 0 | 0 |
| Gadid | 0 | 0 | 0 |
| Pipefish | 0 | 0 | 0 |
| Invertebrates | 0 | n/a | 0 |
| No. with prey | 6 | 4 | - |
| No. additional invertebrates | 0 | n/a | - |
| Total birds sampled | 6 | - | - |

**Appendix S4. Time activity budgets for all common guillemots included in this study.**

|  |  |  |  | **Time budgets (hours/day)** | | | | **Adult energy estimations (kJ/day)** | |
| --- | --- | --- | --- | --- | --- | --- | --- | --- | --- |
| **Year** | **Bird** | **24 hour Section** | **No. dives/day** | **Nest** | **Flight** | **Sea** | **Forage** | **Daily Energy Expenditure** | **Daily Energy Intake** |
| 1999 | 1 | 1 | 65 | 16.0 | 0.3 | 5.1 | 2.6 | 1428 | 1842 |
|  | 2 | 1 | 93 | 11.8 | 0.9 | 7.6 | 3.7 | 1632 | 2105 |
|  | 2 | 2 | 150 | 9.0 | 1.3 | 7.4 | 6.4 | 1690 | 2180 |
|  | 3 | 1 | 197 | 12.8 | 0.3 | 6.2 | 4.7 | 1440 | 1858 |
|  | 4 | 1 | 267 | 11.9 | 0.9 | 6.7 | 4.5 | 1602 | 2066 |
|  | 4 | 2 | 370 | 3.5 | 1.0 | 10.7 | 8.8 | 1684 | 2173 |
|  | 5 | 1 | 249 | 3.0 | 0.6 | 12.9 | 7.5 | 1672 | 2157 |
|  | 6 | 1 | 319 | 11.6 | 0.8 | 5.5 | 6.2 | 1502 | 1938 |
| 2002 | 7 | 1 | 112 | 14.0 | 1.2 | 5.7 | 3.1 | 1550 | 1999 |
|  | 8 | 1 | 105 | 15.4 | 0.7 | 3.1 | 4.8 | 1515 | 1954 |
|  | 9 | 1 | 101 | 12.7 | 0.2 | 7.0 | 4.1 | 1316 | 1698 |
|  | 9 | 2 | 187 | 14.2 | 0.5 | 3.7 | 5.6 | 1466 | 1891 |
|  | 10 | 1 | 196 | 9.9 | 1.2 | 8.0 | 4.9 | 1581 | 2039 |
|  | 10 | 2 | 98 | 13.8 | 0.8 | 6.5 | 2.9 | 1440 | 1858 |
|  | 11 | 1 | 211 | 16.3 | 0.5 | 2.9 | 4.3 | 1459 | 1882 |
|  | 11 | 2 | 235 | 13.4 | 0.6 | 4.9 | 5.1 | 1471 | 1898 |
|  | 12 | 1 | 169 | 10.1 | 0.3 | 11.1 | 2.5 | 1229 | 1586 |
|  | 13 | 1 | 89 | 11.8 | 0.2 | 10.2 | 1.9 | 1183 | 1526 |
|  | 14 | 1 | 157 | 7.9 | 0.9 | 8.0 | 7.2 | 1574 | 2031 |
|  | 14 | 2 | 178 | 9.0 | 0.8 | 7.7 | 6.5 | 1517 | 1957 |
|  | 15 | 1 | 93 | 15.0 | 0.8 | 5.5 | 2.6 | 1442 | 1860 |
|  | 16 | 1 | 171 | 9.7 | 0.8 | 7.5 | 6.0 | 1521 | 1962 |
|  | 17 | 1 | 317 | 7.9 | 2.0 | 5.4 | 8.7 | 1932 | 2493 |
|  | 17 | 2 | 175 | 12.4 | 1.4 | 4.0 | 6.2 | 1715 | 2213 |
| 2003 | 18 | 1 | 131 | 10.6 | 0.6 | 8.1 | 4.7 | 1420 | 1832 |
|  | 19 | 1 | 106 | 14.9 | 0.7 | 5.7 | 2.7 | 1409 | 1817 |
|  | 20 | 1 | 131 | 12.2 | 1.7 | 5.5 | 4.5 | 1691 | 2182 |
|  | 21 | 1 | 125 | 16.4 | 0.4 | 1.9 | 5.3 | 1480 | 1910 |
|  | 22 | 1 | 218 | 7.5 | 1.4 | 6.5 | 8.5 | 1725 | 2225 |
|  | 23 | 1 | 151 | 12.2 | 0.7 | 2.8 | 8.2 | 1634 | 2108 |
|  | 24 | 1 | 110 | 15.6 | 1.0 | 2.7 | 4.7 | 1596 | 2059 |
| 2005 | 25 | 1 | 75 | 17.0 | 0.8 | 3.1 | 3.1 | 1493 | 1927 |
| **Mean** |  |  | **167** | **11.9** | **0.8** | **6.2** | **5.1** | **1532** | **1976** |
| **SD** |  |  | **76** | **3.5** | **0.4** | **2.6** | **1.9** | **150** | **194** |

**Appendix S5. Time activity budgets for all razorbills included in this study.**

|  |  |  |  | **Time budgets (hours/day)** | | | | **Adult energy estimations (kJ/day)** | |
| --- | --- | --- | --- | --- | --- | --- | --- | --- | --- |
| **Year** | **Bird** | **24 hour Section** | **No. dives/day** | **Nest** | **Flight** | **Sea** | **Forage** | **Daily Energy Expenditure** | **Daily Energy Intake** |
| 1999 | 1 | 1 | 33 | 22.9 | 0.5 | 0.5 | 0.2 | 754 | 954 |
|  | 2 | 1 | 314 | 13.3 | 2.3 | 5.3 | 3.0 | 1315 | 1665 |
|  | 2 | 2 | 334 | 11.4 | 1.1 | 7.9 | 3.6 | 1225 | 1551 |
|  | 2 | 3 | 525 | 11.0 | 1.6 | 6.4 | 5.0 | 1233 | 1561 |
|  | 3 | 1 | 164 | 11.8 | 1.8 | 8.3 | 2.1 | 1324 | 1676 |
|  | 3 | 2 | 175 | 16.0 | 2.1 | 4.4 | 1.5 | 1287 | 1629 |
|  | 4 | 1 | 283 | 16.4 | 1.2 | 3.1 | 3.3 | 1137 | 1439 |
|  | 5 | 1 | 243 | 17.3 | 0.9 | 3.0 | 2.9 | 1108 | 1403 |
|  | 5 | 2 | 403 | 16.6 | 0.7 | 2.8 | 3.9 | 1075 | 1361 |
|  | 5 | 3 | 610 | 0.2 | 1.6 | 13.3 | 8.9 | 1326 | 1679 |
| 2005 | 6 | 1 | 749 | 14.2 | 0.7 | 4.9 | 4.2 | 1084 | 1373 |
| 2006 | 7 | 1 | 276 | 15.9 | 3.0 | 1.4 | 3.7 | 1370 | 1734 |
|  | 7 | 2 | 384 | 7.5 | 3.4 | 9.1 | 4.0 | 1331 | 1686 |
|  | 7 | 3 | 313 | 7.9 | 2.6 | 10.3 | 3.3 | 1213 | 1535 |
|  | 7 | 4 | 160 | 10.9 | 1.4 | 9.8 | 2.0 | 1059 | 1341 |
|  | 8 | 1 | 404 | 10.7 | 2.6 | 5.0 | 5.7 | 1329 | 1683 |
|  | 8 | 2 | 270 | 18.5 | 1.6 | 0.2 | 3.7 | 1230 | 1558 |
|  | 8 | 3 | 740 | 4.5 | 1.7 | 11.1 | 6.7 | 1172 | 1484 |
|  | 9 | 1 | 282 | 15.5 | 2.6 | 2.6 | 3.3 | 1308 | 1656 |
|  | 9 | 2 | 293 | 13.7 | 2.8 | 3.7 | 3.8 | 1331 | 1686 |
|  | 10 | 1 | 257 | 15.8 | 2.0 | 2.6 | 3.7 | 1249 | 1582 |
|  | 10 | 2 | 521 | 1.7 | 2.0 | 11.9 | 8.3 | 1237 | 1567 |
|  | 11 | 1 | 290 | 16.2 | 1.8 | 1.2 | 4.8 | 1268 | 1606 |
|  | 11 | 2 | 614 | 6.9 | 2.4 | 7.1 | 7.7 | 1324 | 1677 |
|  | 11 | 3 | 418 | 12.8 | 1.6 | 6.2 | 3.4 | 1161 | 1470 |
|  | 11 | 4 | 716 | 6.7 | 1.2 | 10.6 | 5.5 | 1103 | 1396 |
| **Mean** |  |  | **376** | **12.2** | **1.8** | **5.9** | **4.2** | **1214** | **1537** |
| **SD** |  |  | **189** | **5.3** | **0.8** | **3.8** | **2.0** | **132** | **168** |

**References**

1. Wanless S, Wright PJ, Harris MP, Elston D (2004) Evidence for a decrease in size of lesser sandeels *Ammodytes marinus* in a North Sea aggregation over a 30-yr period. Mar Ecol Prog Ser 279: 237-246.

2. Hilton GM, Furness RW, Houston DC (2000) A comparative study of digestion in North Atlantic seabirds. J Avian Biol 31: 36-46.

3. Croll DA, McLaren E (1993) Diving metabolism and thermoregulation in common and thick-billed murres. J Comp Physiol B 163: 160-166.

4. Birt-Friesen WL, Montevecchi WA, Cairns DK, Macko SA (1989) Activity-specific metabolic rates of free-living northern gannets and other seabirds. Ecology 70: 357-367.

5. Pennycuick CJ (1987) Flight of auks (Alcidae) and other northern seabirds compared with southern Procellariiformes: ornithodolite observations. J Exp Biol 128: 335-347.

6. Penncuick CJ (1997) Actual and ‘optimum’ flight speeds: field data reassessed. J Exp Biol 200: 2355-2361.

7. Bryant DM, Furness RW (1995) Basal metabolic rates of North Atlantic Seabirds. Ibis 137: 219-226.

8. Hislop JRG, Harris MP, Smith JGM (1991) Variation in the calorific value and total energy content of the lesser sandeel (*Ammodytes marinus*) and other fish preyed on by seabirds. J Zool 224: 501–517.

9. Daunt F, Wanless S, Greenstreet SPR, Jensen H, Hamer KC, et al. (2008) The impact of sandeel fishery closure on seabird food consumption, distribution and productivity in the northwestern North Sea. Can J Fish Aquat Sci 65: 362-381.

10. Enstipp MR, Daunt F, Wanless S, Humphreys EM, Hamer KC, et al. (2006) Foraging energetics of North Sea birds confronted with fluctuating prey availability. In: Boyd I, Wanless S, Camhuysen CJ, editors. Top predators in marine ecosystems: Their role in monitoring and management, Conservation Biology 12. Cambridge: Cambridge University Press. pp. 131-142.

11. Drent RH, Daan S (1980) The prudent parent: energetic adjustments in avian breeding. Ardea 68: 225-252.

12. Ricklefs RE, Konarzewski M, Daan S (1996) The relationship between basal metabolic rate and daily energy expenditure in birds and mammals. Am Nat 147: 1047-1071.

13. Bech C, Langseth I, Moe B, Fyhn M, Gabrielsen GW (2002) The energy economy of the arctic-breeding kittiwake (*Rissa tridactyla*): a review. Comp Biochem Physiol A Physiol 133: 765-770.

14. Videler JJ (2005) Avian Flight. Oxford: Oxford University Press. 276 p.

15. Elliott KH, Ricklefs RE, Gaston AJ, Hatch SA, Speakman JR et al. (2013) High flight costs, but low dive costs, in auks support the biomechanical hypothesis for flightlessness in penguins. PNAS 110: 9380-9384.

16. Elliott KH, Le Vaillant M, Kato A, Speakman JR, Ropert-Coudert Y (2012) Accelerometry predicts daily energy expenditure in a bird with high activity levels. Biol Lett 9: 20120919.

17. Birkhead TR, Nettleship DN (1987) Ecological relationships between common murres, *Uria aalge*, and thick-billed murres, *U. Lomvia*, at the Gannet Islands, Labrador: I Morphometrics and timing of breeding. Can J Zool 65: 1621-1629.

18. Harris MP, Wanless S (1988) Measurement and seasonal changes in weight of guillemots *Uria aalge* at a breeding colony. Ring and Migr 9: 32-36.

19. Gaston AJ, Hipfner JM (2006) Body mass changes in Brünnich’s guillemots *Uria lomvia* with age and breeding stage. J Avian Biol 37: 101-109.

20. Elliott KH, Jacobs SR, Ringrose J, Gaston AJ, Davoren GK (2008) Is mass loss in Brünnich’s guillemots *Uria lomvia* an adaptation for improved flight performance or improved dive performance? J Avian Biol 39: 619-628.

21. Tremblay Y, Cherel Y, Oremus M, Tveraa T, Chastel O. (2003) Unconventional ventral attachment of time-depth recorders as a new method for investigating time budget and diving behaviour of seabirds. J Exp Biol 206: 1929-1940.

22. Cairns DK, Bredin KA, Montevecchi WA (1987). Activity budgets and foraging ranges of breeding common murres. Auk 104: 218-224.

23. Cairns DK, Montevecchi WA, Birt-Friesen VL, Macko SA (1990) Energy expenditures, activity budgets, and prey harvest of breeding common murres. Stud Avian Biol 14: 84-92.

24. Monaghan P, Walton P, Wanless S, Uttley JD, Burns MD (1994) Effects of prey abundance on the foraging behavior, diving efficiency and time allocation of breeding guillemots *Uria aalge*. Ibis 136: 214-222.

25. Benvenuti S, Dall’Antonia L, Lyngs P (2001) Foraging behaviour and time allocation of chick-rearing razorbills *Alca torda* at Græsholmen, central Baltic Sea. Ibis 143: 402-412.

26. Dall’Antonia L, Gudmundsson GA, Benvenuti S (2001) Time allocation and foraging pattern of chick-rearing razorbills in northwest Iceland. Condor 103: 469-480.

27. Paredes R, Jones IL, Boness DJ (2006) Parental roles of male and female thick-billed murres and razorbills at the Gannet Islands, Labrador. Behaviour 143: 451-481.

28. Paredes R, Jones IL, Boness DJ, Tremblay Y, Renner M (2008) Sex specific differences in diving behaviour of two sympatric Alcini species: thick-billed murres and razorbills. Can J Zool 86: 610-622.
